# Multi-omics profiles refine L-dopa decarboxylase (DDC) as a reliable biomarker for prognosis and immune microenvironment of clear cell renal cell carcinoma

**DOI:** 10.3389/fonc.2022.1079446

**Published:** 2022-12-05

**Authors:** Kun Chang, Jiaqi Su, Chuanyu Li, Aihetaimujiang Anwaier, Wangrui Liu, Wenhao Xu, Yuanyuan Qu, Hailiang Zhang, Dingwei Ye

**Affiliations:** ^1^ Department of Urology, Fudan University Shanghai Cancer Center, Shanghai, China; ^2^ Department of Oncology, Shanghai Medical College, Fudan University, Shanghai, China; ^3^ Shanghai Genitourinary Cancer Institute, Shanghai, China; ^4^ Department of Neurosurgery, Affiliated Hospital of Youjiang Medical University for Nationalities, Baise, China

**Keywords:** l-DOPA decarboxylase, amino acids metabolism, tumor microenvironment, clear cell renal cell carcinoma (ccRCC), prognosis, biomarker

## Abstract

**Background:**

Increasing evidence indicates that L-dopa decarboxylase (DDC), which mediates aberrant amino acid metabolism, is significantly associated with tumor progression. However, the impacts of DDC are not elucidated clearly in clear cell renal cell carcinoma (ccRCC). This study aimed to evaluate DDC prognostic value and potential mechanisms for ccRCC patients.

**Methods:**

Transcriptomic and proteomic expressions of and clinical data including 532 patients with ccRCC (The Cancer Genome Atlas RNA-seq data), 226 ccRCC samples (Gene Expression Omnibus), 101 ccRCC patients from the E-MTAB-1980 cohort, and 232 patients with ccRCC with proteogenomic data (Fudan University Shanghai Cancer Center) were downloaded and analyzed to investigate the prognostic implications of DDC expression. Cox regression analyses were implemented to explore the effect of DDC expression on the prognosis of pan-cancer. The "limma" package identified the differentially expressed genes (DEGs) between high DDC subgroups and low DDC groups. Functional enrichments were performed based DEGs between DDC subgroups. The differences of immune cell infiltrations and immune checkpoint genes between DDC subgroups were analyzed to identify potential influence on immune microenvironment.

**Results:**

We found significantly decreased DDC expression in ccRCC tissues compared with normal tissues from multiple independent cohorts based on multi-omics data. We also found that DDC expression was correlated with tumor grades and stages.The following findings revealed that lower DDC expression levels significantly correlated with shorter overall survival (P <0.001) of patients with ccRCC. Moreover, we found that DDC expression significantly correlated with an immunosuppressive tumor microenvironment, higher intra-tumoral heterogeneity, elevated expression of immune checkpoint CD274, and possibly mediated malignant behaviors of ccRCC cells via the PI3k/Akt signaling pathway.

**Conclusion:**

The present study is the first to our knowledge to indicate that decreased DDC expression is significantly associated with poor survival and an immune-suppressive tumor microenvironment in ccRCC. These findings suggest that DDC could serve as a biomarker for guiding molecular diagnosis and facilitating the development of novel individual therapeutic strategies for patients with advanced ccRCC.

## Introduction

Renal cell carcinoma (RCC) is the third most common genitourinary malignancy worldwide (1, 2). In 2022, it is estimated that 79,000 new cases are diagnosed as RCC and 13,920 related deaths in the United States (3). Pathologically, RCC incorporates three main subtypes, including clear cell RCC (ccRCC), papillary RCC, and chromophobe RCC (4). Clear-cell RCC is the most common type of RCC with high aggressiveness, accounting for approximately 80% of all RCC pathology types (5). Around 30% of RCC patients are diagnosed as having advanced RCC, and the five-year survival rate is 23% (6). Hence, there is an urgent need to discover the underlying mechanisms of high invasiveness and high metastatic potential to find more reliable biomarkers that could assist in diagnosing and predicting prognosis.

Metabolic reprogramming is widespread in malignant tumors, the most well-known of which is glucose metabolic reprogramming that is termed the “Warburg effect” (7). This inefficient form of energy metabolism supplies the need for new proliferating cancer cells and enables the use of intermediate products to yield biomolecules, such as amino acids, and nucleotides (8). Previous studies revealed that amino acids could have impacts on cell proliferation, the tumor microenvironment, epigenetic modification, and drug resistance (9–14). Previous studies also revealed that amino acid aberrant metabolism was associated with tumor progression and immune infiltration in ccRCC and other cancers (15–18). Therefore, to better understand the profound mechanisms, studies are in demand to identify key amino acid metabolism-related genes and transfer them to drug targets.

L-dopa decarboxylase (DDC) locates at chromosome 7p and encodes a protein that catalyzes the decarboxylation process of L-3,4-dihydroxyphenylalanine (DOPA), L-5-hydroxytryptophan, and L-tryptophan to dopamine, serotonin, and tryptamine, respectively (19). Our previous proteomic analysis demonstrated that L-dopa decarboxylase was significantly downregulated in ccRCC (15). The regional dopamine of the kidney plays a potential role in regulating blood pressure, and the dysregulation of DDC might lead to hypertension, which is a common symptom of RCC (20). Tremmel et al. found that DDC was a favorable prognostic factor in breast cancer (21). However, in prostate cancer, the higher expression of DDC was associated with advanced stages, higher Gleason scores, biochemical recurrence, and short disease-free survival (DFS) (22). Also, the role of DDC has been investigated in the development of colorectal cancer (23), small cell lung cancer (24), and gastric cancer (25). However, the prognostic value and underlying mechanism caused by aberrant L-dopa decarboxylase expression have not been systematically elucidated in ccRCC.

In this study, we thoroughly performed DDC-related bioinformatics analysis in ccRCC and validated conclusions using external cohorts from multi-omics data. We found the downregulation of DDC in ccRCC was significantly associated with worse outcomes. Furthermore, DDC expression showed close relationships with clinicopathologic features and prognosis. We also revealed that DDC was correlated with immune cell infiltration and expressions of immune checkpoint genes. In order to boost the knowledge of basic cancer biology, our study sought to identify the underlying mechanisms of DDC in carcinogenesis and provided a new therapeutic target for ccRCC patients.

## Materials and methods

### Patients’ inclusion and data preprocessing

Proteogenomic expression data of 232 Chinese paired ccRCC and normal samples and 93 ccRCC tumors were included from our institute, the Fudan University Shanghai Cancer Center (FUSCC-ccRCC cohort) (15), and the Clinical Proteomic Tumor Analysis Consortium (CPTAC) (https://proteomics.cancer.gov/programs/cptac). Transcriptomic expression profiles, tumor somatic mutations, and corresponding clinical information of 532 patients with ccRCC and patients across 33 cancers were obtained from The Cancer Genome Atlas (TCGA) database. Transcriptomics data of 226 ccRCC and 196 normal kidney samples were also enrolled from the Gene Expression Omnibus (GEO) database, including GSE36895 (53 ccRCC and 23 normal samples), GSE40435 (101 ccRCC and 101 normal renal samples), and GSE53757 (72 ccRCC and 72 normal samples) cohorts. Additionally, RNA sequences and clinicopathological data of 101 ccRCC patients from the E-MTAB-1980 cohort were available from the ArrayExpress (https://www.ebi.ac.uk/arrayexpress/) database as a transcriptomics validation cohort. Besides, we also included 232 ccRCC samples with proteomics information and available clinical and pathologic data from the FUSCC-ccRCC cohort as a proteomics validation cohort. The details about the FUSCC-ccRCC cohort and how amino acid metabolism clusters are defined were discussed in the previous study (15).

### DDC expression and correlations with clinicopathological features

The DDC expressions of two proteomic cohorts and three transcriptomic cohorts were used to determine whether DDC expression was dysregulated in ccRCC using the Wilcox test. Statistical analyses were conducted on the relationship between DDC expression and clinicopathological features using ggplot2 (v3.3.2) in R software. The Sankey plot of clinicopathological features was conducted in R software.

### Differentially expressed genes identification and functional enrichment analysis

We divided the TCGA cohort into two subgroups based on the median value of DDC expression in order to keep the classification model simple and ensure universality. Then the DEGs between two subgroups were identified with the threshold of |log2(Fold Change)| >1.5 and adjusted P <0.05 using the R package “limma” (26) in the TCGA cohort. The Cluster Profiler package (version: 3.18.0) in R software was employed to analyze the Gene Ontology (GO)-identified functions of potential targets and perform Kyoto Encyclopedia of Genes and Genomes (KEGG) pathway enrichment analysis between subgroups. For pathway analysis, the R software GSVA package was used, choosing parameter as method = ‘ssgsea’ (27). The correlation between DDC expression and pathway score was assessed using Spearman’s correlation analysis.

### Evaluation of immune cells abundance in the TME and immunological response of ccRCC

To evaluate the absolute proportion of tumor-infiltrating lymphocytes (TILs) in ccRCC, we conducted the CIBERSORT and assessed the proportion of all TILs using support vector regression. Besides, to assess the reliability of the algorithms, we used the “immuneeconv” and “pheatmap” R packages that provide an integrated P-value from the six latest algorithms, including TIMER, xCell, MCP-counter, CIBERSORT, EPIC, and quanTIseq for individuals (28). We also explored the TIL differences between two DDC subgroups. The potential therapeutic response to immune checkpoint inhibitors (ICIs) was predicted with the TIDE algorithm, as described previously (29).

### Survival analysis

The primary endpoint was overall survival (OS), and the secondary endpoint was progression-free survival (PFS) in ccRCC patients. Survival curves were performed to assess the prognostic significance using the Kaplan–Meier method and log-rank test with 95% confidence intervals (95% CI). The cut-off value was defined *via* the “survminer” R package or median threshold according to samples assigned to the TCGA cohort. To detect the independent prognostic indicators, we assessed the hazard ratio (HR) and 95% CI using univariate and multivariate Cox logistic regression analysis and visualized the results in the forest plots. We utilized two external validation cohorts, including E-MTAB-1980 and the FUSCC-ccRCC cohort, to confirm the prognostic value of DDC in ccRCC.

### Immunohistochemical analysis

Samples were embedded in paraffin at a thickness of 4 nm. Deparaffinization and rehydration were performed on each slide. Immunohistochemical (IHC) assay was conducted with anti-DOPA Decarboxylase/DDC antibody (1:1,000, ab211535, Abcam) diluted 1:1,000. After incubating the HRP-labeled secondary antibody for 2 h, we performed immunodetection the next day, following the manufacturer’s protocols. Based on the integration of the degree of intensity and density of staining, two independent pathologists evaluated the overall IHC score (from 0 to 12) as follows: negative staining, 0 to 3; positive staining, 4 to 12, as previously described (30).

### Statistical analysis

For statistical analyses, the SPSS software (version 23.0), GraphPad Prism software (version 8.0), or R software (version 3.3.2) were employed. The relationships between DDC expression and clinicopathological characteristics were evaluated using the Chi-square test. The Student’s t-test was used to compare the differences between the two groups. A one-way ANOVA was performed to compare the differences among multiple groups. All hypothesis tests were two-sided, and P-values below 0.05 were regarded as significant.

## Results

### Identification of DDC expression in regulating amino acids metabolism of ccRCC

**Figure 1:**
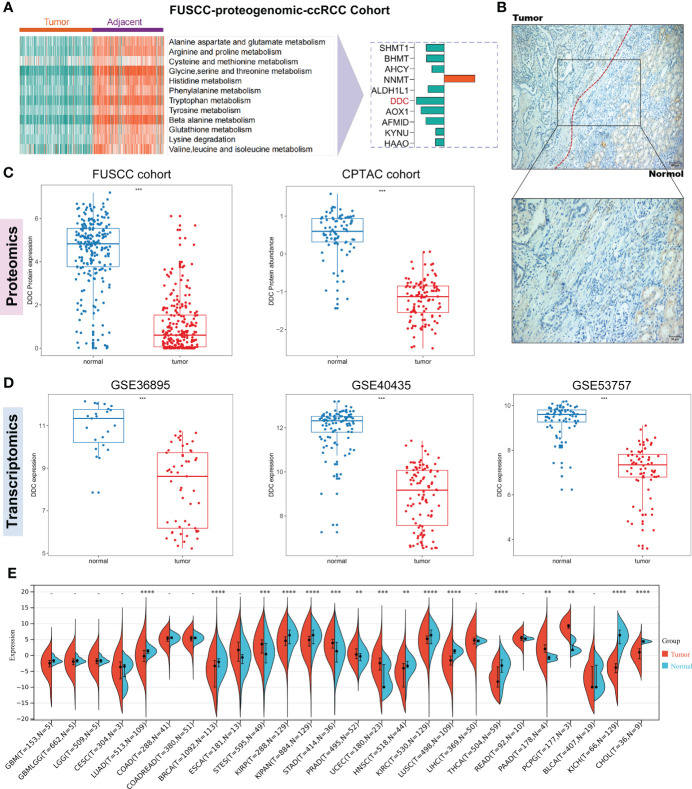
Identification of DDC expression in regulating amino acid metabolism of ccRCC. **(A)** The FUSCC proteomic ccRCC cohort demonstrates that amino acid metabolism is extensively dysregulated between tumor and normal tissue and the DDC protein is significantly downregulated in ccRCC. **(B)** Representative immunohistochemical (IHC) staining of DDC protein in normal kidney and ccRCC tissues. **(C)** Proteomic cohorts (FUSCC and CPTAC) showed DDC protein is lower in tumors than in normal tissue. **(D)** Transcriptomic cohorts (GSE36859, GSE40435, and GSE53757) showed DDC mRNA is lower in tumors than in normal tissue. **(E)** Pan-cancer analysis of DDC mRNA expression in human cancers. CPTAC, Clinical Proteomic Tumor Analysis Consortium; ccRCC, clear cell renal cell carcinoma; DDC, L-dopa decarboxylase; mRNA, messenger RNA (**P <.01; ***P <.001; ****P <.0001).

Our previous study found that tumor and adjacent normal tissue had significant differences in amino acid metabolism-related pathways in the FUSCC proteomic ccRCC cohort (**Figure 1A**). The amino acid metabolism-related proteins, including SHMT1, BHMT, AHCY, ALDH1L1, DDC, AOX1, AFMID, KYNU, and HAAO, were downregulated in ccRCC compared to adjacent normal tissue, while NNMT was upregulated in ccRCC compared to adjacent normal tissue (**Figure 1A**). Thus, we found that DDC was significantly downregulated compared to other downregulated amino acid metabolism-related genes. The immunohistochemistry staining demonstrated a similar phenomenon (**Figure 1B**). To determine whether DDC is aberrantly expressed in ccRCC, we utilized two proteomic cohorts (FUSCC and CPTAC) and three transcriptomic cohorts (GSE36859, GSE40435, and GSE53757) to verify DDC expression at the transcription and translation level. The results demonstrated that both the protein and mRNA levels of DDC were lower in the ccRCC specimen compared to adjacent normal tissue (P <0.001) (**Figures 1C, D**). We next explored DDC expression in human cancers and found that DDC is widely differentially expressed in pan-cancer analysis using the TCGA expression profiling (**Figure 1E**), which indicated that DDC is expressed differently in different human cancers. The aberrant DDC expressions deserved further investigation to determine whether DDC could serve as a therapeutic target.

### Associations between DDC and clinicopathological features in ccRCC from the TCGA cohort

**Figure 2:**
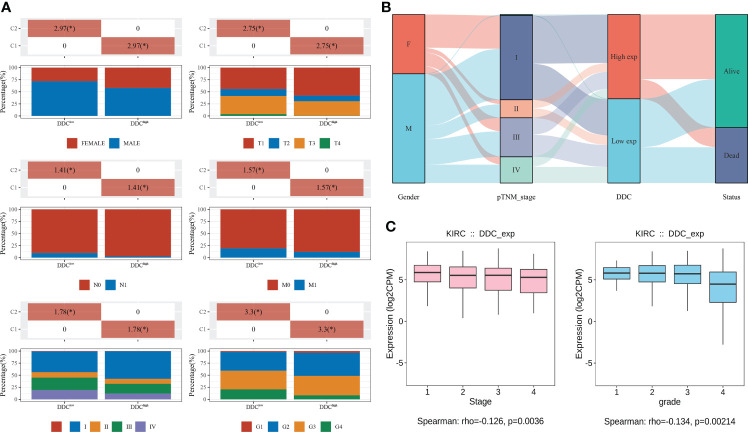
Associations between DDC and clinicopathological features in ccRCC from the TCGA cohort. **(A)** The differences between clinicopathological features and DDC subgroups. **(B)** The distribution of clinicopathological features, DDC subgroups, and live status in KIRC. **(C)** The Spearman correlation between DDC mRNA expression and tumor stage (left) and tumor grade (right) in KIRC. DDC, L-dopa decarboxylase; mRNA, messenger RNA; KIRC, Kidney renal clear cell carcinoma (*P  <.05).

To explore whether DDC expression altered in the process of tumor progression, we divided TCGA cohort into two subgroups based on the median value of DDC expression (DDC^High^ vs. DDC^Low^). We found that different DDC subgroups had different compositions of clinicopathological features, indicating that DDC expression had potential associations with clinicopathological features, including gender, T stage, N stage, and M stage, as well as the American Joint Committee on Cancer (AJCC) stage and tumor grade (P <0.05) (**Figure 2A**). Then, the distribution of clinical phenotypes and DDC expression of the TCGA cohort was presented in **Figure 2B**. Patients diagnosed as stages III–IV were more likely to have lower DDC expression, and the DDC^Low^ group showed a worse prognosis compared to the DDC^High^ group (**Figure 2B**). We then found that DDC expression demonstrated weak but statistically significant correlations with tumor AJCC stage (R = −0.126, P = 0.0036) and tumor grade (R = −0.134, P = 0.00214) (**Figure 2C**). The results indicated the indispensable role of DDC expression in the ccRCC progression process.

### Low DDC expression in ccRCC is associated with worse prognosis

**Figure 3:**
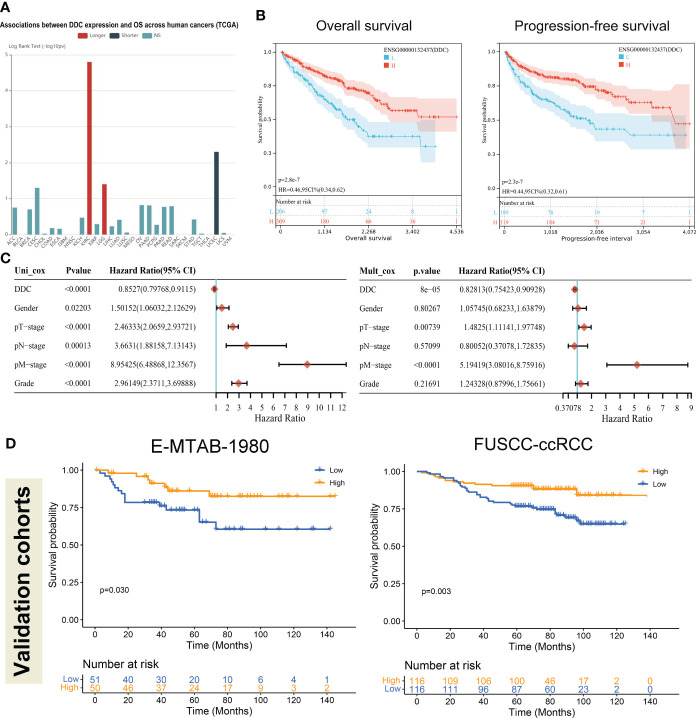
Low DDC expression in ccRCC correlated with a worse prognosis. **(A)** The pan-cancer associations between DDC expression and OS in human cancers. **(B)** Kaplan–Meier survival analysis of the relationships between DDC mRNA expression and OS (P <.001) and PFS (P <.001) in the KIRC cohort. **(C)** Univariate and multivariate Cox logistic regression analysis of OS in the TCGA cohort. **(D)** External Kaplan–Meier survival analysis of the relationships between DDC expression and OS in the E-MTAB-1980 cohort (P = .030) and FUSCC-ccRCC cohort (P = .003). DDC, L-dopa decarboxylase; mRNA, messenger RNA; OS, Overall Survival; PFS, Progression-free Survival; KIRC, Kidney renal clear cell carcinoma.

Due to DDC expression dysregulation in human cancers, we first explored the prognostic value of DDC in pan-cancer analysis. We found that, among all the cancers in the TCGA database, the prognostic implications of DDC expression showed the most significant value in the ccRCC (**Figure 3A**). The following analyses performed in ccRCC cohort demonstrated similar results: lower DDC expression was associated with shorter OS and progression-free survival (PFS) (P <0.001) (**Figure 3B**). We next employed univariate and multivariate Cox analyses to identify the independent prognostic factor. In univariate Cox analysis, the gender, T stage, N stage, M stage, and tumor grade was correlated with worse outcome, while DDC expression was correlated with better outcome (P <0.001). After adjusting for the confounding factors, only DDC expression (HR: 0.828, 95% CI: 0.754–0.909) and M stage (HR: 5.194, 95% CI: 3.080–8.759) could serve as independent prognostic factors (P <0.001) **(** **Figure 3C**). To confirm the prognostic ability of DDC expression, we performed survival analysis in two external cohorts. The results revealed that the lower level of DDC expression was correlated with a worse prognosis in the E-MTAB-1980 cohort (P = 0.030) and in the FUSCC-proteomic-ccRCC cohort (P = 0.003), respectively (**Figure 3D**). The findings above indicated the stable prognostic value of DDC expression, suggesting that DDC expression could be an independent biomarker in predicting outcomes.

### Functional enrichments of DDC expression subgroups

**Figure 4:**
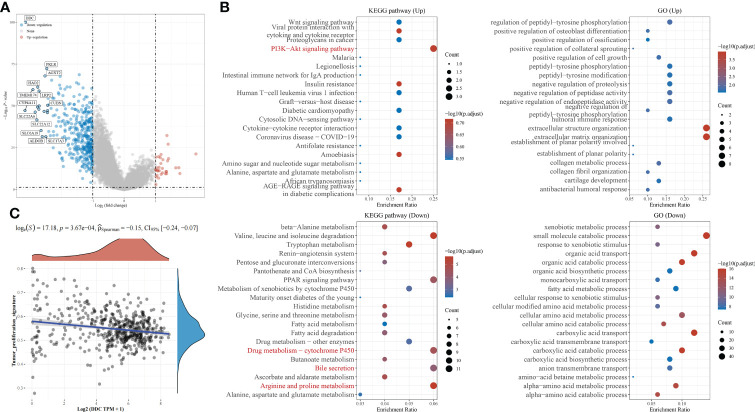
Functional enrichment analysis of DEGs between DDC subgroups. **(A)** The volcano plot of DEGs between DDC subgroups in KIRC. **(B)** The KEGG pathways and GO functional enrichment analysis of DEGs between DDC subgroups. **(C)** The Spearman correlation between DDC expression and tumor proliferation signature (R = −0.15; P <.001). DDC, L-dopa decarboxylase; KIRC, Kidney renal clear cell carcinoma; GO, Gene Ontology; KEGG, Kyoto Encyclopedia of Genes and Genomes.

Based on the above results that DDC expression was lower in ccRCC specimens and correlated with a worse prognosis, we tried to undermine the potential mechanisms that might contribute to the differential prognosis. The differentially expressed genes (DEGs) between the DDC^Low^ and DDC^High^ subgroups are presented in **Figure 4A**. With the exception of DDC, other genes such as PKLR, AGX12, HAO2, TMEM174, LRP2, CYP4A11, CUBN, SLC22A6, SLC22A12, SLC6A19, ALDOB, and SLC17A3 also showed significant low expression in the DDC^Low^ group (**Figure 4A**). The DEGs were used to perform the following functional enrichment analysis: The upregulated DEGs are mainly enriched in the PI3K-Akt signaling pathway, while the downregulated DEGs are mainly enriched in valine, leucine, and isoleucine degradation, the PPAR signaling pathway, drug metabolism-cytochrome P450, bile secretion, and arginine and proline metabolism (**Figure 4B**). The GO results demonstrated that upregulated DEGs were mainly enriched in extracellular structure organization, extracellular matrix organization, and so on. The downregulated DEGs were mainly enriched in small molecule catabolic processes, organic acid transport, organic acid catabolic processes, cellular amino acid metabolic processes, carboxylic acid transport, carboxylic acid catabolic processes, and so on (**Figure 4B**). Because the KEGG pathway is enriched in the PI3K-Akt signaling pathway, we explored the correlation between the tumor proliferation signature and DDC expression. The Spearman’s correlation test indicated potential correlations between DDC and cancer cell proliferation (R = −0.15, P <0.001) (**Figure 4C**). These results revealed the biological differences between the DDC^Low^ and DDC^High^ subgroups and the potential correlation of DDC on proliferation.

### Differential immune microenvironment between DDC expression subgroups

**Figure 5:**
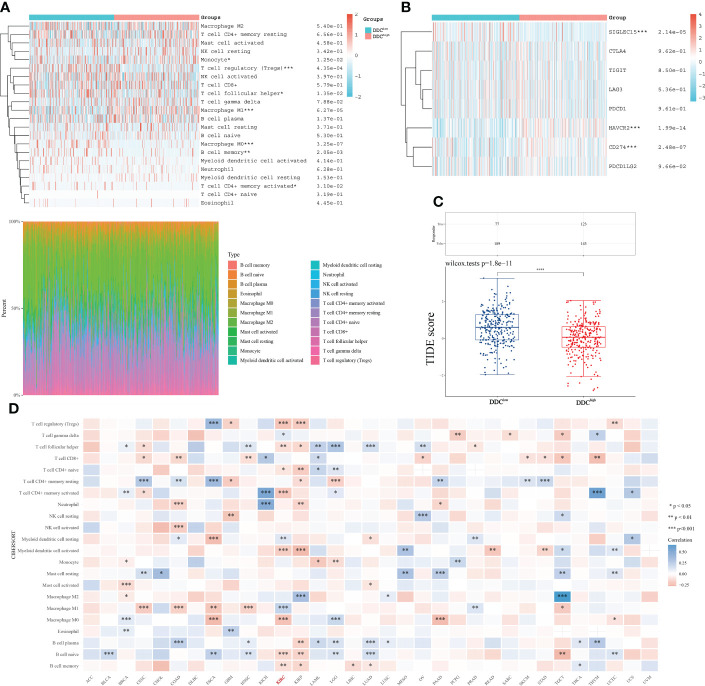
DDC expression correlated with immune microenvironment regulation in ccRCC. **(A)** The differences in immune cell infiltration between DDC subgroups. **(B)** The differences of ICP genes between DDC subgroups. **(C)** The differences in TIDE scores between DDC subgroups. The TIDE score is significantly higher in the DDC^Low^ group (P <.0001). **(D)** The pan-cancer correlations between immune cell infiltrations and DDC expression. DDC, L-dopa decarboxylase; ICP, immune checkpoint; TIDE, Tumor Immune Dysfunction and Exclusion (*P <.05; **P <.01; ***P <.001; ****P <.0001).

Based on the above subgroups, we wondered whether DDC could exert a potential influence on immune cell infiltrations and expressions of ICP genes. The immune cell infiltrations analyzed by the “CIBERSORT” package showed that the proportions of monocytes and M1 macrophages were higher in the DDC^High^ subgroup, while the proportions of Tregs, follicular helper T cells, M0 macrophages, and memory B cells were higher in the DDC^Low^ subgroup (**Figure 5A**). This might partly explain the survival difference in that the prognosis of the DDC^Low^ subgroup was better than that of the DDC^Low^ subgroup. Next, we found that the ICP genes, including SIGLEC15, HAVCR2, and CD274 (PD-L1), expressed differently in DDC subgroups (**Figure 5B**). The SIGLEC15 expression was lower in the DDC^High^ subgroup, while HAVCR2 and CD274 were higher in the DDC^High^ subgroup (P <0.001), which suggested the potential capability in immune regulation. The tumor immune dysfunction and exclusion (TIDE) score has confirmed its ability to predict the immune checkpoint inhibitor (ICI) response (31). In our study, we found that the DDC^High^ subgroup had a lower level of TIDE score than the DDC^Low^ subgroup (P <0.0001) (**Figure 5C**), which meant that the DDC^Low^ subgroup seemed to have a worse immunotherapy response and worse prognosis. To investigate the impacts of DDC protein on immune cell infiltrations, we explored the pan-cancer analysis and found that DDC expression was closely correlated with immune cell infiltrations in the ccRCC cohort (**Figure 5D**). Consistent with the above results, DDC demonstrated significant correlations with M0 and M1 macrophages in ccRCC, and the underlying regulatory mechanisms need to be elucidated in the future.

## Discussion

Kidney cancer is a highly genetically heterogeneous malignant tumor, which may cause patients from different races and regions to carry different gene mutations and genetic phenotypes, which in turn cause the biological behavior of tumor cells and different sensitivity to treatment (32, 33). Therefore, molecular characteristics and subtypes based on multi-omics data are essential for improving treatment efficacy and promoting the achievement of precision medicine in cancer (34, 35). Although there is a growing interest in the function of amino acid metabolism-related genes in cancer, little is known about how DDC proteins work in ccRCC, and it is uncertain whether DDC expression may be used as diagnostic or prognostic markers. Here, we assessed the diagnostic and prognostic value of DDC mRNA and protein expression in external ccRCC cohorts and found potential associations between DDC expression and clinicopathological features. We also explored functional analysis and found aberrant enrichment in the PI3K-Akt signaling pathway. Analysis of immune cell infiltration and ICP expression revealed the underlying regulatory effects of DDC on the tumor microenvironment (TME) and immune system.

In our study, we discovered that DDC mRNA and protein expression were downregulated in ccRCC compared to adjacent normal tissue. There were potential correlations between DDC expression and higher grade, advanced stages. The survival analysis from external validation cohorts revealed that low DDC expression correlated with worse OS. The results above indicate that DDC expression level might be a reliable biomarker assisting in diagnosis and predicting prognosis in ccRCC. To further investigate the potential functions of DDC, we employed KEGG and GO analyses. The findings reveal that DDC protein could possibly enrich the PI3K-Akt signaling pathway, amino acid metabolism, extracellular matrix organization, and so on. The following subgroup analysis identified Treg as being significantly upregulated in the DDC^High^ subgroup, while M1 macrophage was significantly upregulated in the DDC^Low^ subgroup. There were significant differences in ICP gene expressions between the two DDC subgroups, which might eventually contribute to the different TIDE scores and prognosis.

DDC expression has been investigated in several malignant tumors. But in contrast, high DDC expressions are found more frequently in high Gleason’s score and advanced stage, and the underlying mechanism could be attributed to that DDC could co-activate androgen receptor (AR)–ligand transcriptional activity without affecting AR protein expression (36, 37). The following research tested whether the DDC enzymatic inhibitor, carbidopa, would suppress prostate cancer cell proliferation (38). Carbidopa could significantly restrict AR transactivation and PSA upregulation. The cell and castrated mice experiments demonstrated significant tumor growth suppression and decreased serum PSA effects of carbidopa. However, in ccRCC, the opposite strategy should be taken into consideration because of the unique genetic backgrounds between prostate cancer and ccRCC. In breast cancer, DDC upregulation was associated with a longer OS. The two breast cancer cells treated with epinephrine demonstrated contrary results in DDC expression and cell viability (21). Although the previous studies provided a novel insight that antitumor treatments could be combined with endocrine-related therapy strategies, targeted therapy combined with immunotherapy has become the first line of treatment for advanced ccRCC patients and ccRCC patients usually demonstrate relatively fixed types of mutations unlike prostate cancer or breast cancer. The underlying correlations between DDC and mutations such as VHL, BAP1, SETD2, and PBRM1 and whether DDC could serve as a new drug target for treating ccRCC patients or boosting immunotherapy response should be investigated in future studies to better guide treatments.

Dopamine (DA), catalyzed by the DDC protein, plays a role in the normal activities of human lives. It is also an important ingredient in orepinephrine and epinephrine (39). Previous studies found that dopamine receptors could be a biomarker for several malignant tumors (40), which highlights the important role of dopamine that depends on DDC activity in carcinogenesis. Chakroborty et al. found that a low dosage of DDC could restrict tumor angiogenesis *via* inhibiting VEGFR phosphorylation and was correlated with growth restriction *in vitro* (41). Dopamine could significantly promote the efficacy of anti-cancer drugs. The replenishments caused a low proliferation rate and metastatic potential that might be attributed to decreased phosphorylation levels of VEGF receptor-2, mitogen-activated protein kinase, and focal adhesion kinase. Angiogenesis is also one of the major characteristics of ccRCC, and several targeted therapies such as sunitinib, axitinib, and other drugs inhibit ccRCC progression by targeting VEGF targets. Supplementation with dopamine or increasing the activity of DDC enzymes may have a synergistic effect in combination with targeted therapy, significantly inhibiting tumor growth and progression. Moreover, a previous study found that mice with daily stress contributed to increased tumor growth compared to those without daily stress, which could be blocked by dopamine replenishment (42). This study also highlighted the importance of the tumor microenvironment in dopamine deletion and high-stress conditions. Dopamine could activate resting effector T cells (Teffs) and suppress regulatory T cells (Tregs) (43, 44). It also affects helper T-cell differentiation, inhibits Treg activation, takes part in antigen presentation processes, and modulates intracellular signaling pathways, suggesting that dopamine plays an important regulatory role in affecting the tumor microenvironment (45). Dopamine improved the efficacy of chemotherapy *in vivo* and *in vitro* experiments by inhibiting the M2 characteristics of tumor-associated macrophages (TAMs) (46). Qin et al. attempted to re-polarize M2 macrophages to M1 macrophages, and they found that dopamine could upregulate M1-polarized markers and downregulate M2-polarized markers, which could transfer the tumor microenvironment from “cold” to “hot” (47, 48). The M1 macrophage exerted anti-tumor effects and correlated with the immunotherapy response (49, 50). PD-L1 expression (CD274) was previously approved by the FDA as a predictive biomarker for ICI (50, 51). Thus, the differentially expressed PD-L1 and different proportions of M1 macrophages may influence the efficacy of immunotherapy. Future studies should focus on the effects of dopamine catalyzed by the DDC protein on the TME and the underlying mechanisms.

The findings of this study contribute to our knowledge of the function of DDC and recognize it as a potential diagnostic and prognostic factor in ccRCC. However, our study has certain limitations. First, although we utilize several external cohorts to validate DDC expression, large cohorts are needed to validate our conclusions. Second, the diagnostic and prognostic significance of DDC expression has been defined, although the underlying processes regulating its expression levels are still unknown. This will be made clearer by additional functional enrichment and annotation analysis. Third, the DDC protein could influence the percentage of M1 macrophages within the tumor microenvironment, and the underlying mechanisms are needed to be explored in future studies.

In conclusion, our study first systematically identified and assessed DDC expression and its potential functions in the regulation of metabolism and tumor microenvironment of ccRCC. DDC might function as a tumor suppressor protein and has been markedly linked to cancer progression and a worse prognosis in ccRCC.

## Data availability statement

The original contributions presented in the study are included in the article/**Supplementary Material**. Further inquiries can be directed to the corresponding authors.

## Author contributions

Conceptualization: KC, JS, CL, and AA. Data curation and formal analysis: KC, WX, JS, AA, WL, and YQ. Funding acquisition: WX, YQ, HZ, and DY. Investigation and methodology: KC, WX, JS, AA, and WL. Resources and software: WL, WX, YQ, HZ, and DY. Supervision: YQ, HZ, and DY. Validation and visualization: WX, WL, KC, and AA. Original draft: KC, JS, and CL. Editing: YQ, HZ, and DY. All authors listed have made a substantial, direct, and intellectual contribution to the work and approved it for publication.

## Funding

This work was supported by grants from the National Natural Science Foundation of China (nos. 81802525 and 82172817), the Natural Science Foundation of Shanghai (no. 20ZR1413100), the Beijing Xisike Clinical Oncology Research Foundation (no. Y-HR2020MS-0948), the National Key Research and Development Project (no. 2019YFC1316005), the Shanghai “Science and Technology Innovation Action Plan” Medical Innovation Research Project (no. 22Y11905100), and the Shanghai Anti-Cancer Association Eyas Project (nos. SACA-CY21A06 and SACA-CY21B01).

## Acknowledgments

We thank all the writers who gave precious advice to this article.

## Conflict of interest

The authors declare that the research was conducted in the absence of any commercial or financial relationships that could be construed as a potential conflict of interest.

## Publisher’s note

All claims expressed in this article are solely those of the authors and do not necessarily represent those of their affiliated organizations, or those of the publisher, the editors and the reviewers. Any product that may be evaluated in this article, or claim that may be made by its manufacturer, is not guaranteed or endorsed by the publisher.
