# Supplementary figures and images for: Multi-omics profiles refine L-dopa decarboxylase (DDC) as a reliable biomarker for prognosis and immune microenvironment of clear cell renal cell carcinoma
Source: Front Oncol. 2022 Dec 5;12:1079446. doi: 10.3389/fonc.2022.1079446 (PMC9760914; doi:10.3389/fonc.2022.1079446)

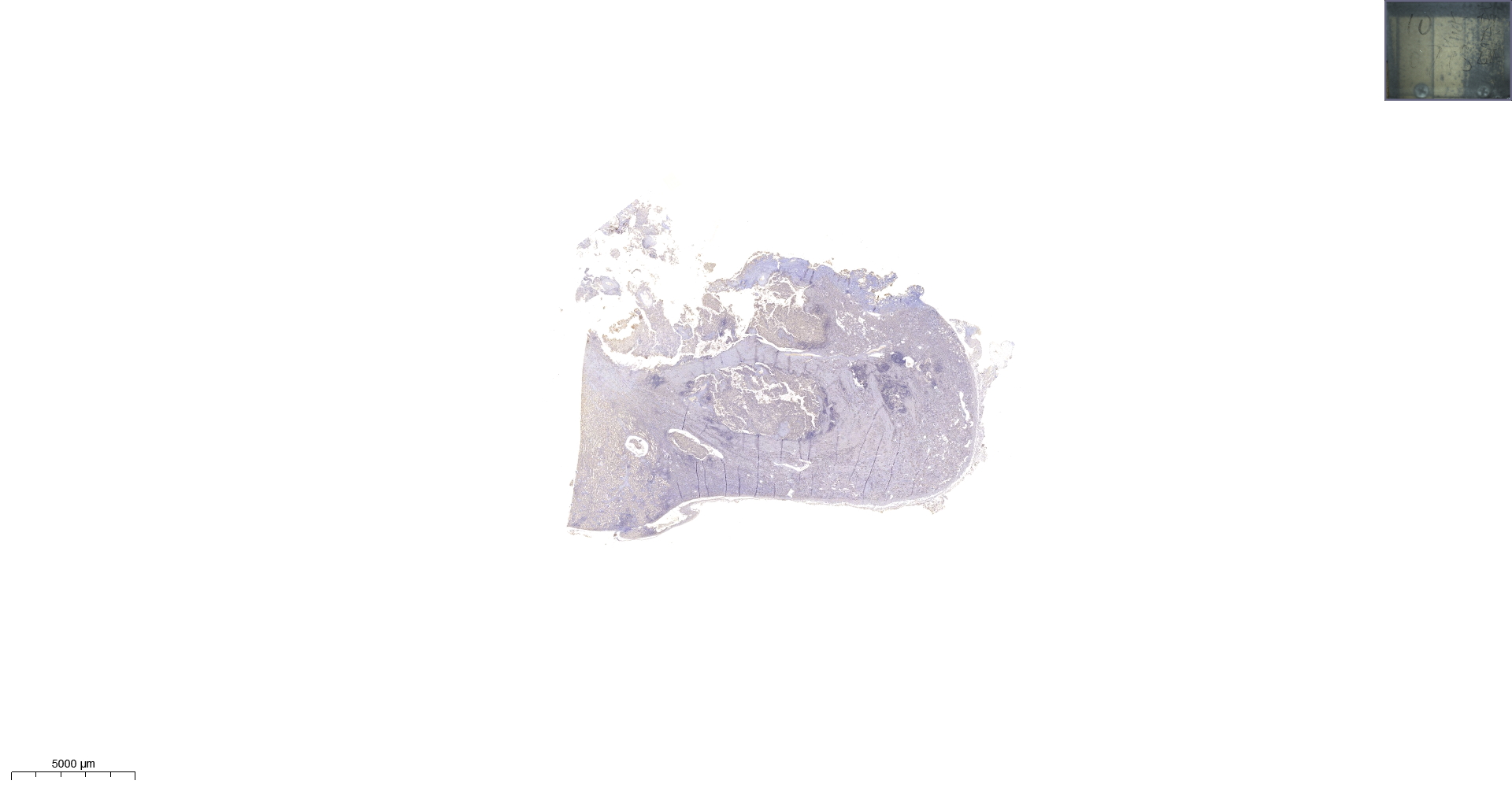

Supplement: Supplementary file 3 [file Image_1.jpeg]

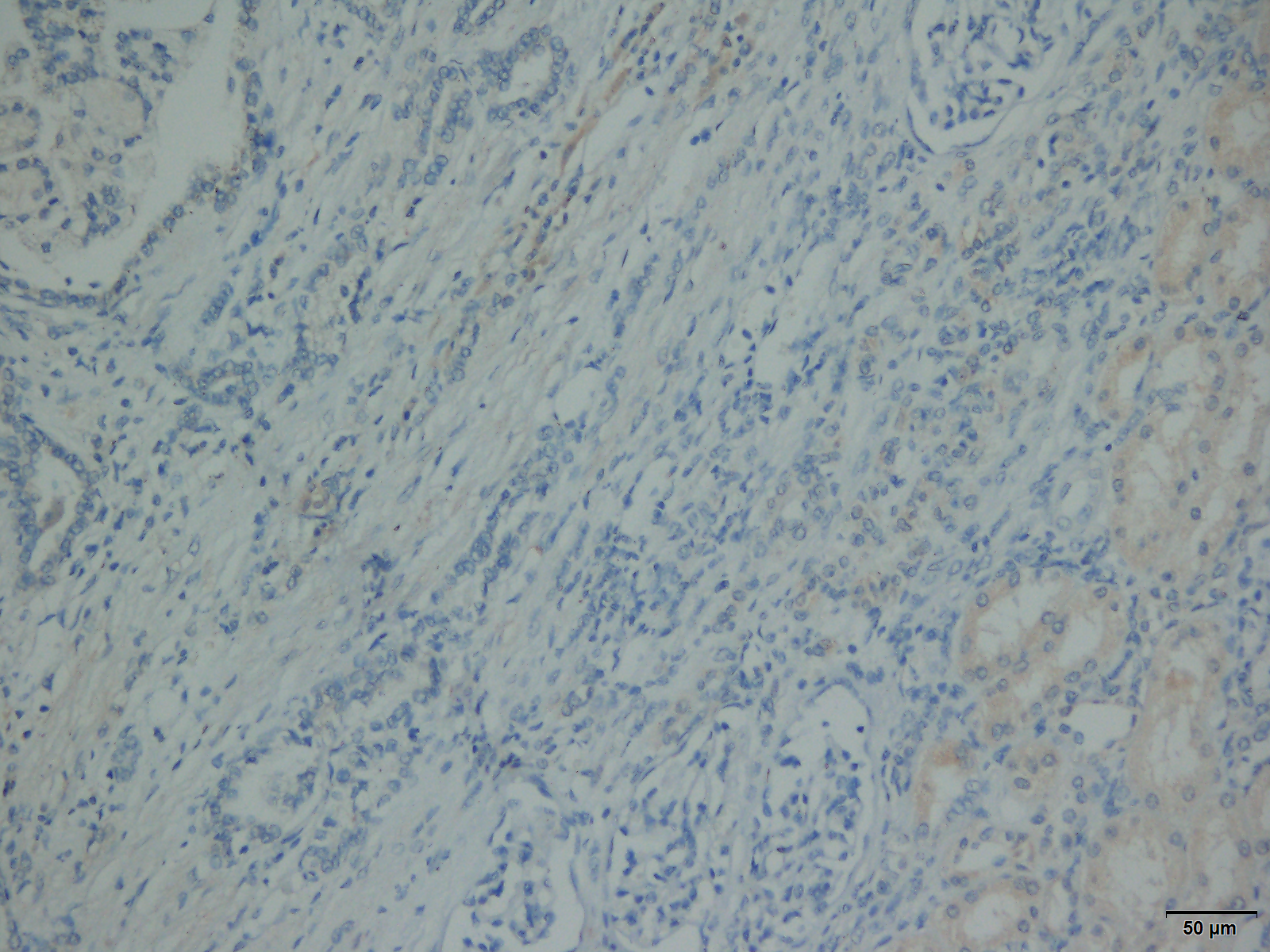

Supplement: Supplementary file 4 [file Image_2.tif]

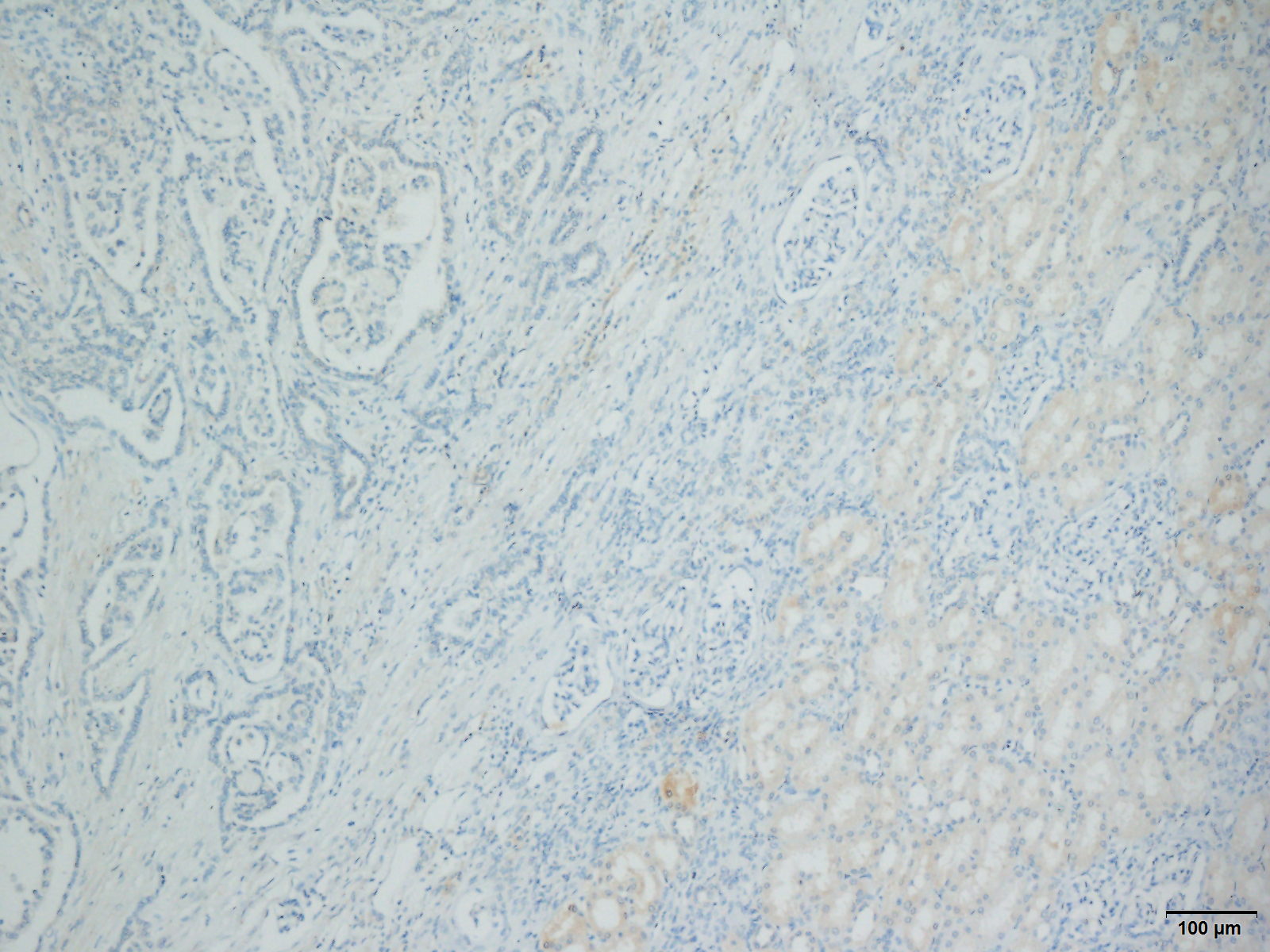

Supplement: Supplementary file 5 [file Image_3.tif]
